# Modeling perchloroethylene degradation under ultrasonic irradiation and photochemical oxidation in aqueous solution

**DOI:** 10.1186/1735-2746-9-32

**Published:** 2012-12-23

**Authors:** Mahdi Kargar, Ramin Nabizadeh, Kazem Naddafi, Simin Nasseri, Alireza Mesdaghinia, Amir Hossein Mahvi, Mahmood Alimohammadi, Shahrokh Nazmara, Bagher Pahlevanzadeh

**Affiliations:** 1 Department of Environmental Health Engineering, School of public Health, Tehran University of Medical Sciences, Tehran, Iran; 2 Center for Air Pollution Research, Institute for Environmental Research, Tehran University of Medical Sciences, Tehran, Iran; 3 Center for Water Quality Research, Institute for Environmental Research, Tehran University of Medical Sciences, Tehran, Iran; 4 Center for Solid Waste Research, Institute for Environmental Research, Tehran University of Medical Sciences, Tehran, Iran; 5 Department of Epidemiology and Biostatistics, School of public Health, Tehran University of Medical Sciences, Tehran, Iran

**Keywords:** Perchloroethylene, Ultrasound, Photochemical oxidation, Kinetics models

## Abstract

Sonolysis and photochemical degradation of different compounds such as chlorinated aliphatic hydrocarbons are among the recent advanced oxidation processes. Perchloroethylene is one of these compounds that has been mainly used as a solvent and degreaser. In this work, elimination of perchloroethylene in aqueous solution by ultrasonic irradiation, andphotochemical oxidation by ultra violet ray and hydrogen peroxide were investigated. Three different initial concentrations of perchloroethylene at different pH values, detention periods, and concentrations of hydrogen peroxide were investigated. Head space gas chromatography with FID detector was used for analyses of perchloroethylene. This research was performed in 9 months from April through December 2011.

Results showed that perchloroethylene could be effectively and rapidly degraded by ultrasonic irradiation, photochemical oxidation by ultra violet ray, hydrogen peroxide and a combination of these methods. Kinetics of perchloroethylene was strongly influenced by time, initial concentration and pH value. Degradation of Perchloroethylene increased with decrease in the initial concentration of perchloroethylene from 0.3 to 10 mg/L at all initial pH. The results showed an optimum degradation condition achieved at pH = 5 but did not affect significantly the perchloroethylene destruction in the various pH values. Kinetic modeling applied for the obtained results showed that the degradation of perchloroethylene by ultrasound and photo-oxidation followed first order and second order model. The percentage of removal in the hybrids reactor was higher than each of the reactors alone, the reason being the role of hydroxyl radical induced by ultrasound and photochemical reaction.

## Introduction

AS the number of substances resistant to biodegradation have increased, and the conventional biological methods were unable to complete the treatment of these materials; Therefore, new technologies are required to degrade these resistant molecules to smaller ones. The smaller molecules can be degraded by biological processes [1]. New technologies include advanced oxidation processes such as Fenton, peroxone, common use of ozone, UV irradiation, hydrogen peroxide and the use of ultrasonic and photo-catalytic oxidation processes [2]. One category of resistant material to biological degradation is chlorinated hydrocarbons. These materials cause water resources contamination and affect human health. Several studies have been carried out in removing various organic materials from water and aqueous solutions [3-6].

Perchloroethylene (PCE) is a chlorinated hydrocarbon that has been mainly used as a solvent in dry cleaning, degreaser in metal parts manufacturing, and as a precursor in the production of chlorofluorocarbons [7,8]. Perchloroethylene is included in products such as motor vehicle cleaners, stain removers, adhesive and wood cleaners [9,10]. It is a volatile, nonflammable and colorless liquid with a stench that has odor threshold of 1 ppm [9]. The summary of PCE physical properties are shown in Table  1[11].

**Table 1:** PCE properties (EPA, 1994)

**Molecular Weight(g/mol)**	**Chemical formula**	**Density at 20°C(g/mL)**	Solubility*at 25°C(mg/L)	**Melting point(°C)**	**Boiling point(°C)**	**Henry**^**’**^**s law constant (atm.m**^**3**^**/mol)**
165.85	C_2_Cl_4_	1.63	150	−22	121	1.8 × 10^-2^

*Solubility in water.

Various applications and inappropriate handling and disposal, results in detection of PCE in groundwater, surface water, wastewater, air and food [12-16]. PCE is considered as a probable carcinogenic chemical (Group 2A) to humans [9]. It has also many other adverse health effects [7-12,17], due to which United State Environmental Protection Agency (US.EPA) has set the maximum contaminant level (MCL) and maximum contaminant level goal (MCLG) for PCE as 0.005 mg/L and zero, respectively [18].

Conventional water and wastewater treatment processes have poor efficiency in removal of chlorinated compounds such as PCE [19]. Advanced processes such as membrane process, granular activated carbon and air stripping are effective for removal of chlorinated compounds but they are expensive and transfer the contamination to another phase [20]. A large number of new technologies have been emerged that include sonochemistry, photochemistry, electrochemistry and combined treatment methods such as reductive dehalogenation and biodegradation for the degradation of chlorinated compounds [19]. Advanced oxidation processes (AOPs) are able to degrade chlorinated compounds such as PCE into less harmful compounds by using a combination of ultraviolet radiation, H_2_O_2_ and ultrasonic waves. Ultrasonic waves are hydroxyl radicals produced during cavitations. Therefore ultrasonic waves are among the advanced oxidation processes [20].

Several studies have been performed on application of photochemical oxidation and sonolysis especially at low concentrations in removal of various pollutants [20-22]; but there are few studies regarding PCE degradation by sonolysis and photochemical oxidation (UVC/ H_2_O_2_) at micromolar concentration and with a 130 kHz frequency ultrasound. In this work, the degradation rates of PCE at different concentration levels and different pH levels with using an ultrasound bath at 130 kHz frequency and photochemical oxidation with UVC/ H_2_O_2_ were studied. Continuous models of PCE degradation were also determined.

## Materials and methods

### Experimental setup

**Figure 1:**
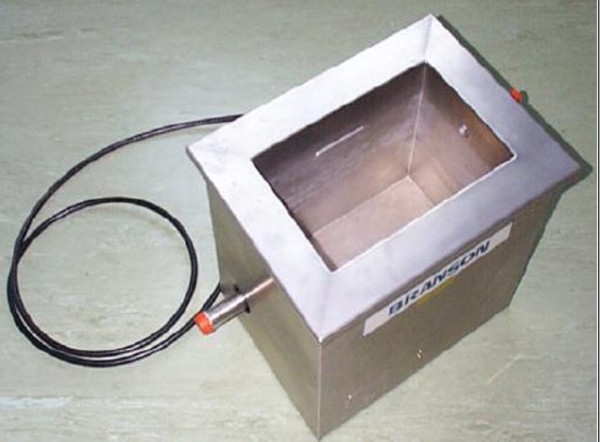
Ultrasonic equipment.

**Figure 2:**
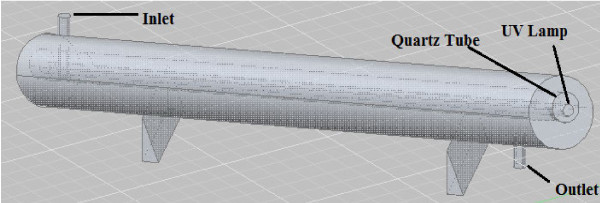
A schematic of UVC equipment.

This experimental research was conducted at the Department of Environmental Health Engineering at Tehran University of Medical Sciences between April and December 2011. Ultrasound bath of the solution in a 300 mL glass reactor (Figure  1) was performed with a 130 kHz frequency and acoustic intensity of 2.5 W/cm^2^ (Table  2). The characteristics of UVC reactor (Figure  2) are shown in the Table  3.

**Table 2:** Characteristics of ultrasound reactor used in the experiments

**Parameters**	**Characteristics**
Frequency	*35-130kHz*
Power	*500W*
Acoustic Intensity	*2.5W/cm*^*2*^
Flow type	*Batch*
Reactor volume	*3.7 Liter*
Dimensions	L = 30cm, W = 25cm, H = 32cm

**Table 3:** Characteristics of UVC reactor used in the experiments

**Parameters**	**Characteristics**
Model	TUV
Company	Philips
Power(Watt)	55(low pressure mercury)
Intensity(W/cm^2^)	52
Wavelength(nm)	253.7
Flow type	*Batch*
Reactor volume	*8 Liter*
Reactor dimensions	d = 15cm, L = 100cm
UV lamp dimensions	d = 20mm L = 90cm

Solutions of different concentrations of PCE (0.30, 3 and 10 mg/L) were prepared by dissolving PCE (Merck Co., Germany) in distilled water. The concentrations of H_2_O_2_ were 10, 50 and 100 mg/L. Temperature was monitored during sonication and maintained constant at 25°C by cooling water. Samples were taken from the ultrasonic and UV reactors at given reaction times (5, 10, 20, 30, 40, 50 and 60 min). The number of samples (regarding pH, time, and concentration as variables) was 63 for each reactor.

### Analytical methods

Analyses were performed by head-space gas chromatography technique. Concentrations of PCE samples were determined through GC-FID analysis (VARIAN CP-3800, Australia). The gas chromatograph was fitted with a CP-Sil 8 CB capillary column (30 m, 0.32 mm ID, 0.25 μm film thickness). The injector temperature was 150°C, initial oven temperature was 35°C (held for 1 min) and increased to 100°C at a rate of 16°C/min, held for 5 min. The inlet (200 μL) was operated in 20% split mode. Helium (99.999%) was used as the carrier gas at 1 mL min^-1^. The lowest detection level (MDL) for PCE analysis by GC with the above mentioned method was 5 μg/L.

## Results and discussion

**Figure 3:**
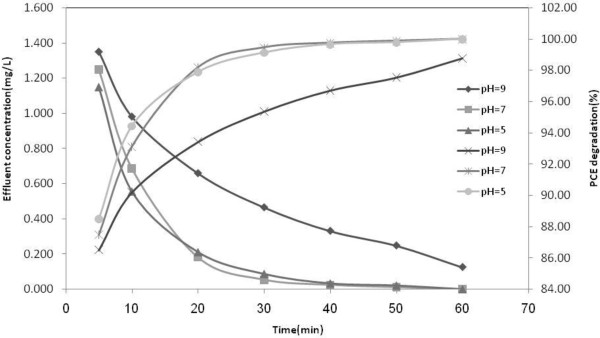
**Degradation of aqueous solution of 10 mg/L at different pH subjected to ultrasound and UVC/H**_**2**_**O**_**2 **_**(100 mg/L); T = 25°C.**

**Figure 4:**
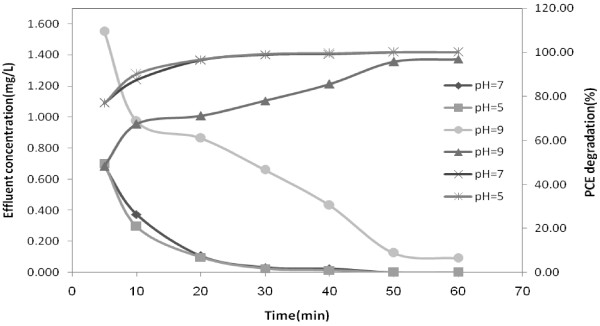
**Degradation of aqueous solution of 0.3 mg/L at different pH subjected to ultrasound and UVC/H**_**2**_**O**_**2 **_**(100 mg/L); T = 25°C.**

**Figure 5:**
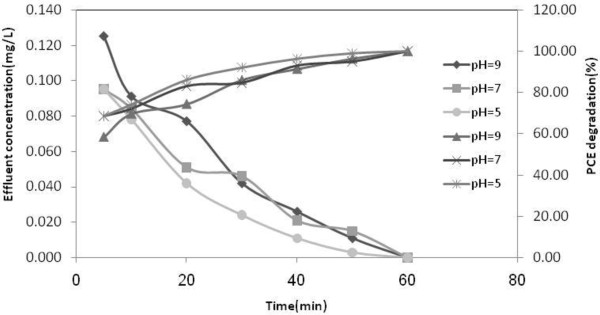
**Degradation of aqueous solution of 3 mg/L at different pH subjected to ultrasound and UVC/H**_**2**_**O**_**2 **_**(100 mg/L); T = 25°C.**

Aqueous solution with initial concentrations of (0.3, 3, and 10 mg/L) for PCE at different pH values were sonicated and photochemically oxidated. The investigation was carried out in six reactors (Table  4). The efficiency at different pH values and kinetic constants in these reactors are illustrated in Tables  5, 6 and 7. The mean removal efficiency in the US/UVC/ H_2_O_2_ reactor at various concentrations of H_2_O_2_ is illustrated in Table  8 and Figures  3, 4, 5, respectively.

**Table 4:** Models of perchloroethylene degradation under various reactors

**Reactor**	**Model of reactor**
US	$$\begin{array}{l} \text{y=}1.725+0.304C_{\mathrm{in}}-0.461pH-0.012Time+0.018\mathrm{int}C_{\mathrm{in}}.pH\\ -0.005\mathrm{int}C_{\mathrm{in}}.Time+0.0001sqTime+0.008sqC_{\mathrm{in}} \end{array}$$
UVC	y = − 3. 5 + 1.31*pH* + 0.029 int *C*_*in*_. *pH* − 0.004 int *C*_*in*_. *Time* − 0.084*sqpH* + 0.035*sqC*_*in*_
US + UVC	$$\begin{array}{l} \text{y=}0.286+0.138C_{\mathrm{in}}-0.0161pH+0.007\mathrm{int}C_{\mathrm{in}}.pH-0.002\mathrm{int}C_{\mathrm{in}}.Time\\ +0.0001sqTime-0.005sqC_{\mathrm{in}} \end{array}$$
UVC + US + H_2_O_2_ 10mg/L	$$\begin{array}{l} \text{y=}0.296+0.454C_{\mathrm{in}}-0.024Time+0.01\mathrm{int}C_{\mathrm{in}}.pH-0.004\mathrm{int}C_{\mathrm{in}}.Time\\ +0.0001sqTime-0.023sqC_{\mathrm{in}} \end{array}$$
UVC + US + H_2_O_2_ 50mg/L	y = 0.324 + 0.269*C*_*in*_ − 0.024*Time* − 0.003 int *C*_*in*_. *Time* + 0.0001*sqTime* − 0.006*sqC*_*in*_
UVC + US + H_2_O_2_ 100mg/L	$$\begin{array}{l} \text{y=}1.253+0.181C_{\mathrm{in}}-0.343pH-0.025Time-0.002i\mathrm{int}C_{\mathrm{in}}.Time\\ +0.029sqpH+0.0001sqTime-0.009sqC_{\mathrm{in}} \end{array}$$

**Table 5:** Mean efficiency and kinetic order degradation of PCE at various pH, subjected to US reactor

**C**_**0**_**(mg/L)**	**pH**	**Efficiency (%)**	**K (1/min)**	**Reaction order**
10	9	57.31	0.0094	First
10	7	64.54	0.0148	First
10	5	65.31	0.0162	First
3	9	58.27	0.0155	Second
3	7	68.56	0.0157	Second
3	5	70.31	0.0184	Second
0.3	9	29.38	0.043	Second
0.3	7	35.57	0.037	Second
0.3	5	39.42	0.0606	Second

**Table 6:** Mean efficiency and kinetic order degradation of PCE at various pH, subjected to UVC reactor

**C**_**0**_**( mg/L)**	**pH**	**Efficiency (%)**	**k(1/min)**	**Reaction order**
10	9	53.81	0.0082	First
10	7	49.65	0.0056	First
10	5	62.58	0.0137	First
3	9	76.15	0.0148	First
3	7	77.16	0.0181	First
3	5	82.61	0.023	First
0.3	9	69.42	0.0287	First
0.3	7	66.42	0.0247	First
0.3	5	74.76	0.0391	First

**Table 7:** Mean efficiency and kinetic order degradation of PCE at various pH, subjected to US/UVC reactor

**C**_**0**_**( mg/L)**	**pH**	**Efficiency (%)**	**k(1/min)**	**Reaction order**
10	9	88.85	0.0194	First
10	7	91.36	0.0221	First
10	5	91.89	0.0215	First
3	9	82.97	0.035	First
3	7	88.057	0.0518	First
3	5	86.67	0.0545	First
0.3	9	71.24	0.0436	First
0.3	7	81.38	0.0339	First
0.3	5	82.05	0.0393	First

**Table 8:** **Mean efficiency degradation of PCE at various pH, subjected to US/UVC/ H**_**2**_**O**_**2 **_**reactor**

**C**_**0**_**( mg/L )**	**pH**	**Efficiency of UVC + US + H**_**2**_**O**_**2 **_**reactor (%)**		
		**With 10mg/L H**_**2**_**O**_**2**_	**With 50mg/L H**_**2**_**O**_**2**_	**With 100mg/L H**_**2**_**O**_**2**_
10	9	81.97	88.97	94.05
10	7	83.79	89.59	96.84
10	5	85.79	93.77	97.06
3	9	65.0	79.2	79.076
3	7	67.83	87.83	94.16
3	5	71.98	88.9	94.6
0.3	9	62.58	78.33	82.28
0.3	7	72.52	83.09	85.5
0.3	5	78.52	85.38	87.95

Regression analysis was used for modeling of perchloroethylene degradation under various reactors. To calculate the effluent concentration and efficiency, the effluent concentration (y), pH (5–9), time (5 to 60 min) and initial concentration ( 0.3 to 10 mg/L) were considered as independent variables in the model (Table  4) for each reactor.

Parameters that had a significant difference were included in the model (Table 4). These parameters include main variables (pH, primary concentration of PCE and time), interaction and square of main variables. For example, in the US reactor pH, initial concentration (C_in_), time, interaction Cin, pH and interaction C_in_, time and square C_in_ and time have a significant difference. These models can be used to calculate the efficiency of those concentrations for which the test was not performed (such as 1 mg/L).

Decomposition of PCE in the ultrasonic reactor with 10 mg/L of concentration, UVC, UVC/US and UVC + US + H_2_O_2_ reactor in all concentrations followed first order kinetics model and in the ultrasonic reactor for 3 and 0.3 mg/L of concentration followed second order kinetics model. Also with increasing the initial concentration of PCE, the apparent first and second order rate constants decreased, indicating non–elementary nature of the photochemical and sonolysis reactions. Most investigators have observed the kinetics of photolysis and sonolysis of pollutants to be first order [23-26].

This dependence of degradation rate constants on initial concentration was similar to other studies [20,23,27]. Degradation rate of PCE at pH = 5 was higher than the other pH levels, but the difference between the other pH values were not significant.

The consumed energy by various reactors for treatment of 1 m^3^ of contaminated water is illustrated in the Table  9. As shown in Table  9 the energy consumption in the hybrid process (UVC + US + H_2_O_2_ 100 mg/L) is the lowest, while the Ultrasonic process has a maximum consumed energy.

**Table 9:** **Consumed energy by various reactors**[28]

**Reactor**	**Concentration(mg/L)**	**Concentration(mg/L)**	**Concentration(mg/L)**
	**0.3**	**0.3**	**0.3**
	**Consumed energy (kw/m**^**3**^**)**		
US	1389	714	595
UVC	153	61	63
US + UVC	420	317	77
UVC + US + H_2_O_2_ 10mg/L	652	451	353
UVC + US + H_2_O_2_ 50mg/L	292	75	30
UVC + US + H_2_O_2_ 100mg/L	25	15	5

The hybrid methods showed higher efficiencies compared to the single reactors. The reactors’ efficiency from high to low are illustrated below:

$$\begin{array}{l} \mathrm{UVC}+\mathrm{US}+H_{2}O_{2}\;10\mathrm{mg}/L\\ \;>\mathrm{UVC}+\mathrm{US}+H_{2}O_{2}\;50\mathrm{mg}/L>\mathrm{UVC}+\mathrm{US}> \end{array}$$

$$\begin{array}{l} \mathrm{UVC}+\mathrm{US}+H_{2}O_{2}\;10\mathrm{mg}/L>\mathrm{UVC}>\mathrm{US} \end{array}$$

## Conclusion

Sonolysis and photochemical degradation of PCE were performed under various experimental conditions such as initial concentration, pH, time of reaction and type of reactor. The reduction of initial concentration of PCE increased the degradation rate of PCE increased and parallel to the increase of initial concentration, the degradation rate constant declined, but the initial pH of the solution did not significantly affect the PCE destruction. It was shown that the application of UVC + US + (H_2_O_2_ 100 mg/L) could effectively remove PCE in 60 minute. Therefore, the mentioned hybrid process can be considered as process for complete removal of PCE in reasonable detention time. Furthermore, Lower energy consumption of the hybrid process compared to the other methods, make it more feasible to be used in full scale PCE removal practice.

## Competing interests

The authors confirm that there is not any competing interest in publishing the results of the study.

## Authors’ contributions

This study is a part of Ph.d thesis of Mr. kargar who collect the laboratory data. The study supervised by Dr. KN and Dr. RN who is the corresponding author and done the technical proofread of the article . Dr. AM, Dr, SN, Dr. HM, and Dr. MA took part as consultant and took part in designing the research. Mr. SN was the GC expert and performed the GC analysis. The statistical analysis and modeling was done by Mr. BP who is statistical expert. The overall implementation of this study including design, laboratory experiments , data analysis, and manuscript preparation was performed by corresponding author and MK. All authors have made extensive contribution into the review and finalization of this manuscript. All authors have read and approved the final manuscript.
